# Seroprevalence of TBEV in bank voles from Poland—a long-term approach

**DOI:** 10.1038/s41426-018-0149-3

**Published:** 2018-08-15

**Authors:** Maciej Grzybek, Mohammed Alsarraf, Katarzyna Tołkacz, Jolanta Behnke-Borowczyk, Beata Biernat, Joanna Stańczak, Aneta Strachecka, Leszek Guz, Klaudiusz Szczepaniak, Jerzy Paleolog, Jerzy M. Behnke, Anna Bajer

**Affiliations:** 1 Department of Tropical Parasitology, Medical University of Gdańsk, Gdańsk, Poland; 2 Department of Parasitology, University of Warsaw, Warsaw, Poland; 3 Department of Forest Pathology, Poznan University of Life Sciences, Poznan, Poland; 4 Department of Biological Basis of Animal Production, University of Life Sciences in Lublin, Lublin, Poland; 5 Department of Biology and Fish Disease, University of Life Sciences in Lublin, Lublin, Poland; 6 Department of Parasitology and Invasive Diseases, University of Life Sciences in Lublin, Lublin, Poland; 7 Department of Zoology, Animal Ecology & Wildlife Management, University of Life Sciences in Lublin, Lublin, Poland; 8 School of Life Sciences, University of Nottingham, Nottingham, NG7 2RD UK

## Abstract

Rodents are known to play a significant role as reservoir hosts for TBEV. During three sequential expeditions at 4-year intervals to three ecologically similar study sites in NE Poland, we trapped bank voles (*Myodes glareolus*) and then tested their blood for the presence of specific antiviral antibodies to TBEV. The strongest effects on seroprevalence were the extrinsic factors, site of capture of voles and year of sampling. Seroprevalence increased markedly with increasing host age, and our analysis revealed significant interactions among these three factors. Seroprevalence did not differ between the sexes. Therefore, based on the seroprevalence results, the dynamics of TBEV infection differ significantly in time, between local sub-populations of bank voles and with increasing host age. To fully understand the circulation of the virus among these reservoir hosts and in the environment, long-term monitoring is required and should employ a multi-site approach, such as the one adopted in the current study.

## Introduction

Rodents, members of the most abundant and diversified mammalian order Rodentia^1^, can pose a significant threat to the health of humans, livestock, and wildlife because they are hosts for a wide range of pathogens and in some cases constitute important reservoir hosts for life-threatening zoonoses^2^.

The tick-borne encephalitis virus (TBEV), the causative agent of tick-borne encephalitis (TBE), is a zoonotic flavivirus in the family Flaviviridae that is endemic throughout the northern Palearctic, spanning an area from central and northern Europe and across Siberia to Japan in the far east^3^. TBEV is maintained in nature in a cycle that includes tick vectors of the *Ixodes persulcatus* complex and their vertebrate hosts. The most important vector in Central Europe is *Ixodes ricinus*^4,5^, and small rodents are the most important hosts for the immature stages of *I. ricinus*^6^. There are five known routes for the transmission and maintenance of TBEV. Ticks become infected when feeding on a viremic host^7^ and maintain the virus via transstadial^8^ or/and transovarial transmission^9^, or through co-feeding on a non-viremic host^10^. Sexual transmission from male to female ticks is also known to occur^11^. Consequently, all hematophagous stages of ticks can transmit the virus to mammalian hosts^12^. Rodents have been considered to play an essential role in maintaining TBEV in nature by carrying persistent latent infections^13,14^.

TBEV is the most important causative agent of arboviral infections in Europe and is responsible for distressing neurologic symptoms in patients^4^. Incidence of the disease has greatly increased over the past decades, growing into a serious human threat, and changes in the spatial distribution of TBE cases have been concurrently observed^15,16^. Therefore, it is essential to identify the endemic areas and to monitor the temporal changes of this virus in order to ensure that suitable preventive measures are implemented successfully by human communities living in or close to current endemic sites. In recent years, since the sudden and as of yet unexplained increase in the incidence of TBEV infection in Poland in 1993, an average of 250 TBE cases each year have been recorded in the country with a mean incidence of 0.75 cases/100,000 people^17^. Incidence of TBEV infection is highest in northeastern regions of Poland, and these areas are considered to be a TBE-hyperendemic region of the country (11.53 cases/100,000 inhabitants)^18^. However, in contrast to other parts of Europe, there is still a gap in our knowledge about the extent of TBEV prevalence in bank voles (*Myodes glareolus*) in Poland, and in the exact role that they play as reservoirs of this virus in the region. Bank voles are one of the most common and widespread rodent species in European forests^19^ and are recognized as among the most important mammalian reservoir hosts of TBEV^14,20^.

We hypothesized that both extrinsic (temporal and spatial) and intrinsic (age) factors play a major role in affecting the seroprevalence of TBEV in bank voles and consider it important to understand the role and relative importance of each of these factors in order to gain a greater insight into the local epidemiology of TBEV infection. In this study, we aimed: (1) to assess the seroprevalence of TBEV infection in bank voles in three geographically separated but ecologically similar study sites in the region and (2) to identify the intrinsic (host age, sex) and extrinsic (year, study site) factors that most affect TBEV seroprevalence in this rodent species. Here, we report the results of our study, which was conducted during three sequential expeditions at 4-year intervals to study sites in the hyperendemic region of the country. Our results are the first to report on the seroprevalence of TBEV in wild rodents from Poland and make an important contribution to European datasets. Our study permits future regional comparative analyses of the extent of this viral agent in *M. glareolus* and the role of this particular host species in maintaining, perpetuating, and disseminating TBEV infections throughout the continent.

## Results

The overall seroprevalence rate of TBEV was 14.8% (12.5–17.5) (Table 1), but this rate varied significantly between surveys (YEAR × PRESENCE/ABSENCE of TBEV antibodies; *χ*^2^_2_ = 24.07; *P* < 0.001) Bank voles sampled in 2006 and 2010 exhibited 2 to 2.5-fold higher seroprevalence rates than those sampled from 2002 (Table 1). The site or location of sampling also had a significant effect (SITE × PRESENCE/ABSENCE of TBEV antibodies; *χ*^2^_2_ = 36.2; *P* < 0.001), with the overall highest seroprevalence rate recorded among bank voles from Pilchy (28.1% [20.2–37.5]). Bank voles collected from the other two sites exhibited lower seroprevalence rates (Urwitałt = 7.8% [5.5–10.8] and Tałty = 11.3% [8.6–14.6]).

**Table 1:** Seroprevalence of TBEV by year, site and host age

Year	Host age				
	Site	*N*	1	2	3
2002	Urwitałt	64	0.0 (0.0–23.8)	0.0 (0.0–13.4)	0.0 (0.0–13.4)
	Tałty	70	4.5 (0.2–22.2)	6.5 (1.9–17.4)	17.6 (5.0–41.7)
	Pilchy	69	0.0 (0.0–17.6)	11.5 (3.2–30.4)	8.3 (1.5–26.7)
Overall by year		203			**5.4 (3.7–7.8)**
2006	Urwitałt	95	7.7 (1.4–24.6)	4.5 (0.7–17.7)	4.0 (0.2–19.6)
	Tałty	65	7.7 (1.4–24.6)	0.0 (0.0–22.2)	12.5 (3.5–31.0)
	Pilchy	67	24.1 (11.5–43.0)	38.5 (16.6–65.8)	76.0 (56.1–89.0)
Overall by year		227			**18.1 (14.7–21.9)**
2010	Urwitałt	86	0.0 (0.0–20.8)	25.0 (12.9–41.9)	11.5 (3.2–30.4)
	Tałty	96	11.5 (3.2–30.4)	13.0 (3.7–32.4)	19.1 (8.8–36.6)
	Pilchy	56	11.8 (2.1–35.0)	50.0 (24.3–75.7)	32.1 (23.2–42.6)
Overall by year		238			**19.7 (16.2–23.8)**
Overall by age			8.7 (4.4–16.0)	13.7 (10.8–17.3)	20.8 (17.2–24.9)
Overall		668			**14.8 (12.5–17.5)**

Seroprevalence is presented as percentage and reported with + /−95% CL

*N* number of bank voles tested, 1 immature juvenile voles, 2 mostly young adult voles, 3 breeding older animals

The TBEV seroprevalence rate was essentially identical in both sexes (males = 14.9% [11.30–19.38] and females = 14.7% [11.21–19.04]) (NS), but differed significantly between host age classes (AGE × PRESENCE/ABSENCE of TBEV antibodies; *χ*^2^_2_ = 13.05; *P* < 0.001). Seropositivity was 2.4-fold higher in the oldest individuals compared to the youngest (Table 1) and was at an intermediate level among age class 2 bank voles.

**Fig. 1:**
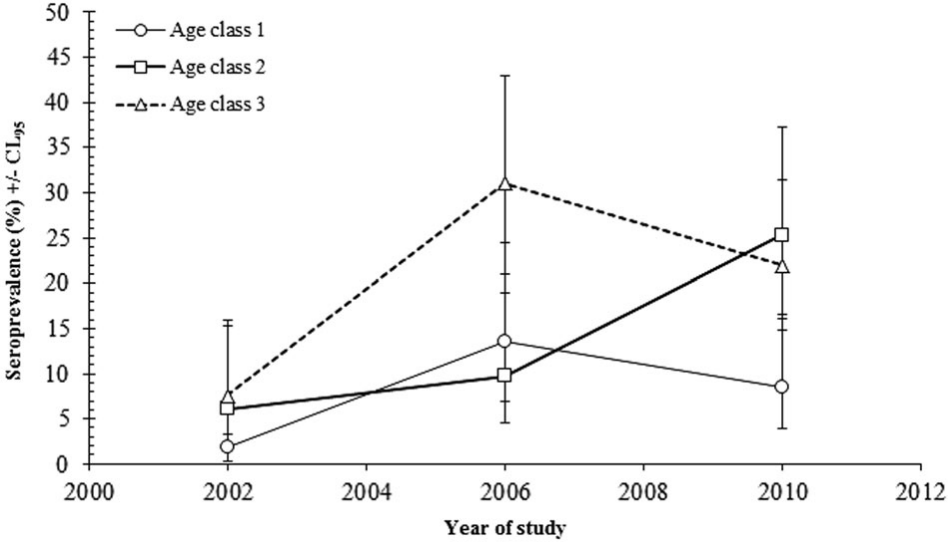
Age related changes in TBEV seroprevalence by year of survey

Although the seroprevalence rate differed significantly between surveys, it was confounded by an interaction with host age (YEAR × AGE × PRESENCE/ABSENCE of TBEV antibodies; *χ*^2^_4_ = 11.43; *P* = 0.022) (Fig. 1). In 2002, the seroprevalence rate of TBEV was lowest in age class 1 bank voles, but much higher or similar in age classes 2 and 3. A similar pattern was recorded in 2010, but in 2006 the pattern was slightly different, with the seroprevalence rate being low and similar in age classes 1 and 2, but much higher in age class 3 (Fig. 1).

**Fig. 2:**
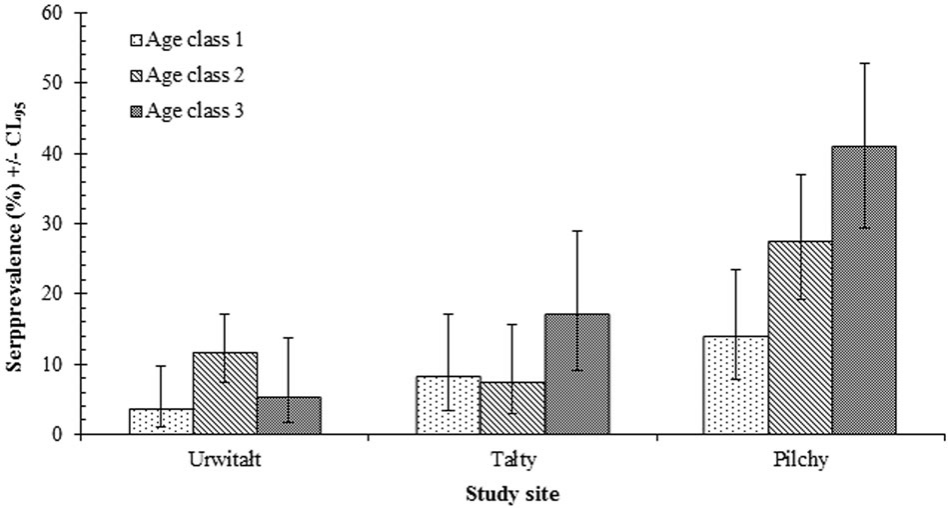
Age related changes in TBEV seroprevalence by the study site

The differences in the seroprevalence rates between sites were also confounded by a significant interaction with host age (SITE × AGE × PRESENCE/ABSENCE of TBEV antibodies; *χ*^2^_4_ = 13.05; *P* = 0.011) (Fig. 2). There was a progressive increase in TBEV seroprevalence rates with the increase in host age among bank voles from Pilchy. The highest seroprevalence rate among bank voles from Talty was also recorded in the oldest voles; but, in Urwitałt, no clear trend was apparent. We also observed a more complex interaction that included two extrinsic and one intrinsic factor (YEAR × SITE × SEX × PRESENCE/ABSENCE of TBEV antibodies; *χ*^2^_4_ = 14.07; *P* = 0.007). However, since seroprevalence rates did not differ significantly between the sexes overall, we did not explore this further.

**Fig. 3:**
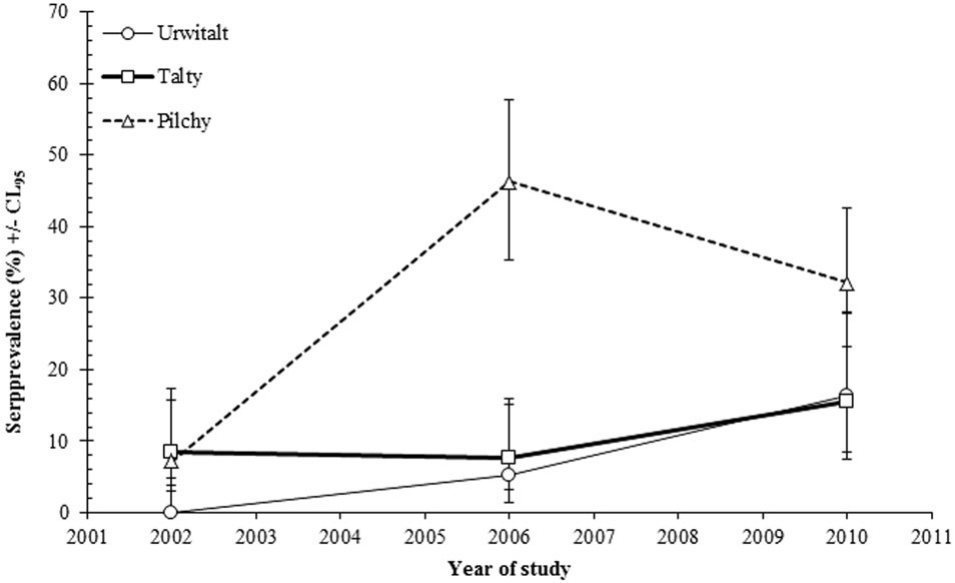
Spatiotemporal dynamics of TBEV seroprevalence within study sites

The spatiotemporal dynamics of seroprevalence rates are illustrated in Fig. 3 (YEAR × SITE × PRESENCE/ABSENCE of TBEV antibodies; *χ*^2^_4_ = 22.6; *P* < 0.001). Interestingly, seroprevalence rates were very similar and stable in bank voles from Tałty throughout the study period, but displayed different dynamics at the other two sites. At Urwitałt, the seroprevalence rate increased slowly but constantly from 0% in 2002 to 16.3% in 2010. At Pilchy, there was a 6.4-fold increase in the TBEV seroprevalence rate, which was as high as 46.3% in 2006 and as low as 32.1% in 2010.

## Discussion

In this paper, we have presented original data on the first study, and to-date the longest recorded period, of serological monitoring of TBEV infections in bank voles in Poland. The Mazury Lake District is a highly endemic region for TBE in Poland (in 2010–2016: the mean incidence was 3.17 cases/100,000 people)^21^, and the average percentage of seropositive patients with neurological symptoms was found to be 15.5% (12.9–18.9) in this region^22^. Our results show high overall seroprevalence rate of TBEV antibodies (14.8%) in *M. glareolus*, one of the most common rodent species in the locality, suggesting an important role for this species as a reservoir host of TBEV in this region. These findings are not only of considerable relevance to public health in the region but could also be important for other European regions populated by *M. glareolus*. They complement earlier reports from Poland and other European countries suggesting frequent infection of *Myodes* (*Clethrionomys)* spp. with TBEV^13,14,23–25^.

Once infected after feeding on viremic rodents, ticks transmit TBEV to new susceptible hosts during subsequent feeds, and continue to harbor the virus until they die^26^. The prevalence of the virus in questing ticks is generally low when compared to the reported seroprevalence rate in rodents, e.g., 0.28% in Scandinavia^27^, 0.24% in Lithuania and 0.11–0.96% in Poland^28,29^. However, TBEV infection in ticks enhances their questing activity^30^. Our previous studies, carried out at the same sites as the current study, showed a very high prevalence rate (80–100%) of tick infestations in woodland and fallow land rodents^31^. Paziewska et al. (2010) also reported a high prevalence rate (81%) of juvenile stages of *I. ricinus* on bank voles and heavy infestations with a high ratio of *I. ricinus* larvae to nymphs in forest rodents in contiguous sites. Although *M. glareolus* may develop resistance to feeding ticks after repeated infestations, these rodent hosts still play a significant role as TBEV reservoirs, alongside *Apodemus* spp^32^., because the virus can persist in bank voles as a latent infection^13,14^. The high prevalence rate of antibodies against TBEV that we detected in bank voles reinforces the idea that they play a role as reservoir hosts for TBEV, and thereby are a source of infection for human communities in the region, and should not be underestimated.

Year-to-year fluctuations in the prevalence and abundance of other pathogens have been well documented in bank voles sampled from our study sites in the past^33–37^. While some pathogen species have fluctuated markedly (e.g., some helminths and hemoparasites) or have even become locally extinct in our study sites, others have shown relative stability from year to year. The temporal dynamics of TBEV infection clearly place this infectious agent among the former group since we found marked temporal variations in seroprevalence rates of TBEV in bank voles. Populations of many rodent species, including bank voles, are also known to fluctuate markedly, exhibiting regular and predictable cycles over several years^38^ but can often fluctuate without a predictable period between peak densities^33,39,40^. Similar and concurrent fluctuations have been observed in their ectoparasite populations^40,41^.

**Fig. 4:**
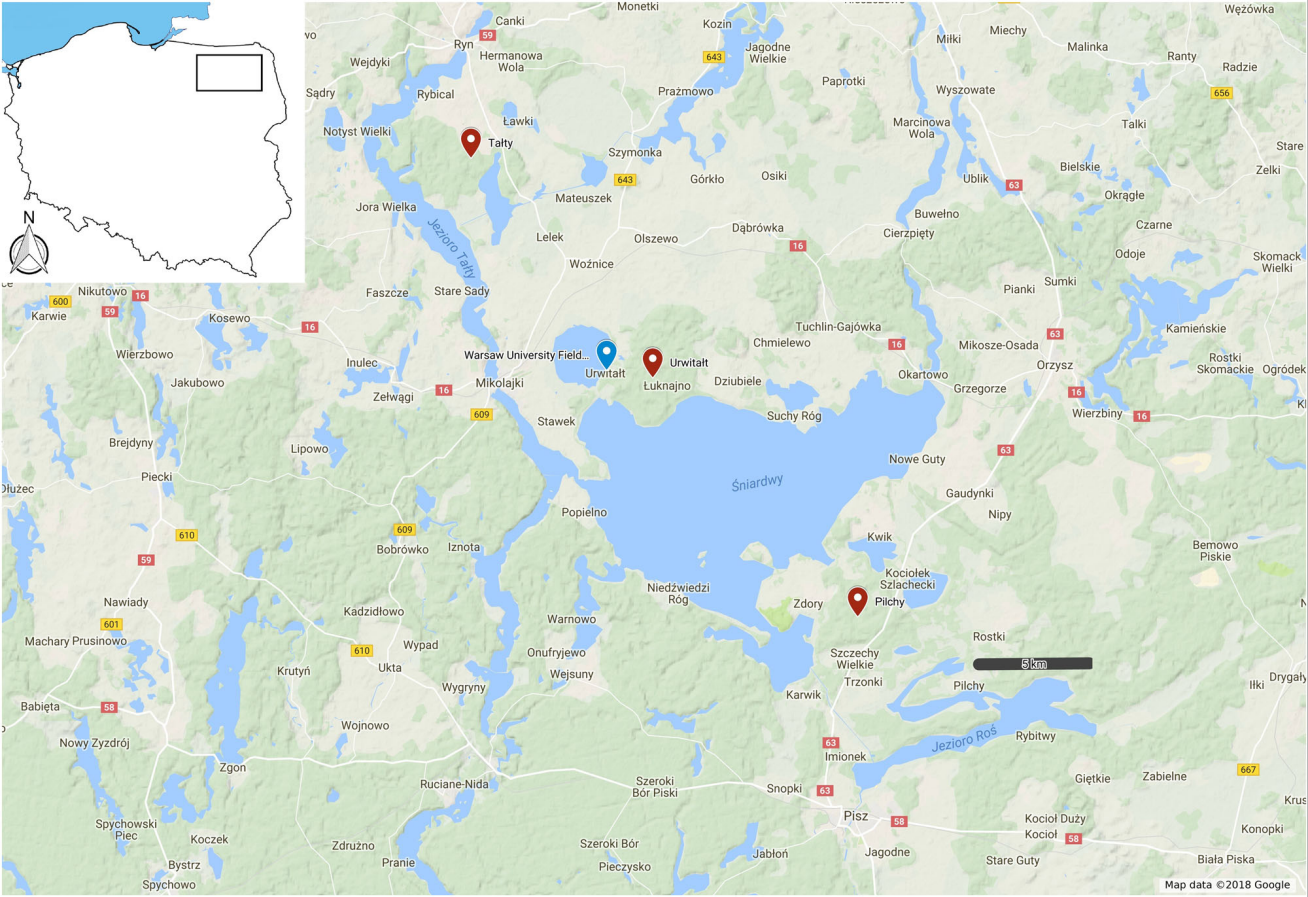
Localizations of the study sites in the Mazury Lake District in N.E. Poland (Google Maps, 2018). Sites are approximately 10 km from one another in a NW-SE transect

We also found significant differences in the seroprevalence TBEV between voles from different sites despite the documented similarity in the ecological structure and relative proximity of our three study sites (Fig. 4). Our current results therefore, complement those that we have previously reported on other pathogens (helminths and hemoparasites), and clearly establish that the site from which host populations are sampled is the most important factor influencing prevalence and abundance of infection. Both parameters can vary markedly when derived from host populations living in different sites within the same geographical region, even when those sites are considered to be ecologically very similar. We originally hypothesized that the dependence of TBEV on tick vectors and that the widespread distribution of ticks in Polish forests where their final deer hosts are also present^42,43^, would essentially tend to negate any differences in these parameters between sites. However, as our data revealed, this turned out not to be the case. Spatial differences in the seroprevalence of TBEV, combined with temporal changes as discussed above, added another level of complexity to the epidemiology of TBEV infection in rodents. Thus, temporal changes in the prevalence were not consistent across sites, and for example, a sharp increase in the prevalence of seropositivity was recorded among bank voles from Pilchy between 2002 and 2006, whereas the seroprevalence rates remained low among bank voles from the other two sites. Therefore, short-term monitoring may be insufficient to fully understand the circulation of the virus within rodent populations. Based on our results, a reliable picture of how a given pathogen is distributed spatially and how it fluctuates temporally in its host population can only be derived from studies utilizing a multi-site approach for monitoring microparasites and macroparasites in a chosen geographical region, applied over many years. Such a long-term approach, with regular sampling of wild rodent populations over a lengthy period of years, is more likely to capture crucial unidirectional as well cyclical changes in prevalence and that will improve our understanding of the epidemiology of TBEV in its rodent reservoirs.

Our present data show that host age also plays an important role, significantly affecting the seroprevalence rate of TBEV. The seroprevalence rate of the virus was higher among mature bank voles compared with younger individuals. In the case of pathogens that cause chronic infections, the likelihood of being infected and the abundance of parasite burdens increase with the age of the host^34^. The current work was based on the presence/absence of specific antibodies against TBEV, and hence positivity in our assay reflected the history of previous infections and not necessarily current infections. Therefore, it was not unexpected to find that older animals were more likely to have experienced infection than juveniles. Bank voles are born in nests and spend most of their time in the nests until they are capable of foraging outside^44,45^, so questing ticks are unlikely to be encountered until they become more mobile and spend more time in the vegetation surrounding nests.

Finally, the results presented in this paper provide a significant and novel contribution to our understanding of the seroprevalence rate of TBEV within bank vole populations. Our data show that the dynamics of TBEV transmission change markedly with time but not always to the same degree in sites in close proximity to one another. Future studies should establish exactly how peaks of infection can be accurately predicted locally. The patchy distribution of seropositivity among bank voles from local subpopulations, as reflected in the between-site differences in the seroprevalence rates that we found, is of fundamental interest, and sample coverage over a wider geographical range would be more informative. Likewise, the relatively low prevalence of the virus in the *I. ricinus* population compared to some other tick-transmitted pathogens (i.e., *Anaplasma phagocytophilum* and *Borrelia burgdorferi*) (Stanczak et al. 2004) is puzzling, given the prevalence of TBEV in mammalian hosts. Future research should focus on resolving the enigma of how the TBEV is maintained for long periods of time despite such a low prevalence in *I. ricinus*. Our study sites are located in a region of Poland that is extremely popular with the tourists and thousands of summer holidaymakers who visit the Mazury Lake District each year. Therefore, the high seroprevalence rate of TBEV in bank voles presents a significant threat to public health, and a capacity to predict peak-years and high risk sites may help to prevent human cases of TBE and thereby contribute significantly to the public health of local populations and visitors to the region.

## Materials and methods

### Ethical approval

This study was carried out in accordance with the recommendations found in the Guidelines for the Care and Use of Laboratory Animals of the Polish National Ethics Committee for Animal Experimentation. Formal permits were obtained, allowing for trapping of animals in the field and for subsequent laboratory analysis of sampled materials. Our project was approved by the First Warsaw Local Ethics Committee for Animal Experimentation.

### Study sites

Our three study sites are located in the Mazury Lake District region in the northeastern corner of Poland (Fig. 4). They are separated by natural barriers, i.e., lakes, and therefore are isolated from one another in ecological time. The host species is panmictic across the region, and genetic studies have revealed that some gene flow exists between the three populations^46^. The sites have been described comprehensively in our earlier paper^47^.

### Collection of bank voles

Bank voles were sampled from mid-August to mid-September in 2002, 2006 and 2010. Trapping was carried out for 3–4 consecutive days at a time at each site. The methods used for trapping rodents and for sampling and processing trapped animals have been thoroughly described by Behnke et al.^36,37,47^. Three age classes were established according to the methods of Behnke et al.^47^ and Grzybek et al.^48^ using principal components analysis of a range of morphological measures including body weight and dried eye lens weight as follows: class 1—immature juvenile bank voles; class 2—mostly young adult bank voles; and class 3—breeding older animals.

Blood samples were collected directly from the heart by cardiac puncture using a sterile 1.5 mL syringe immediately after death from over-exposure to an anesthetic. Blood was allowed to clot at room temperature. After separation of the blood clot, samples were centrifuged at 5000 rpm for 10 min using an MPW High-Speed Brushless Centrifuge. Serum was collected and stored at −80 °C until the samples could be analyzed upon completion of the fieldwork.

### Immunochemical analysis by ELISA

We carried out ELISAs for the quantitative determination of anti-TBE-IgG antibodies using the IMMUNOZYM® FSME (TBE) IgG All Species Kit (PROGEN Biotechnik GmbH, Germany) and according to the manufacturer’s instructions. In total, we analyzed 668 bank vole sera. The optical density was measured at a wavelength of 450 nm (0.1 s) using a PerkinElmer Victor 3 Multilabel Plate Counter. Calculation of anti-TBE-IgG concentration was performed quantitatively using the reference curve. The optical density at 450 nm was transformed into Vienna units (VIEU). Samples were scored as negative for anti-TBE-IgG antibodies if VIEU/mL was <63, as borderline if VIEU/mL was in the range of 63–126, and as positive if VIEU/mL was >126. All borderline samples were tested twice, and if the second test confirmed a borderline score for a sample, it was treated subsequently as negative. Otherwise, previously borderline samples were considered to be positive or negative according to the value of the score derived on retesting.

### Statistical analysis

Seroprevalence values (percentage of seropositive animals) are given with 95% confidence limits in parenthesis (CL_95_) or error bars on figures and were calculated by a bespoke software based on the tables of Sokal and Rohlf, (1995)^49^.

The statistical approach has been documented comprehensively in our earlier publications^36,37,47,50^. For analysis of seroprevalence rates, we used maximum likelihood techniques based on log-linear analysis of contingency tables in the software package IBM SPSS Statistics Version 21 (IBM Corporation). This approach is based on categorical values of the factors of interest, which are used to fit hierarchical log-linear models to multidimensional cross-tabulations using an iterative proportional-fitting algorithm and detects associations between the factors, one of which may be presence/absence of anti-TBE-IgG antibodies against the TBE virus. Initially, full factorial models were fitted, incorporating as factors sex (2 levels: males and females), age (3 levels), year (3 levels: 2002, 2006, and 2010), and site (3 levels: Urwitałt, Tałty, and Pilchy). The presence or absence of anti-TBE-IgG antibodies against the TBE virus (seroprevalence rate) was considered as a binary factor. All these five factors were fitted initially to all models that were evaluated. For each level of analysis, beginning with the most complex model involving all possible main effects and interactions, those combinations that did not contribute significantly to explaining variation in the data were eliminated stepwise beginning with the highest level interaction (backward selection procedure). A minimum sufficient model was then obtained, for which the likelihood ratio of *χ*^2^ was not significant, indicating that the model was sufficient in explaining the data. The importance of each term in interactions involving seroprevalence in the final model was assessed by the probability that its exclusion would alter the model significantly and these values are given in the text. The remaining terms in the final model that did not include seroprevalence (for example, variation among sites in the number of animals of each sex sampled [site × sex]) are not given but can be made available from the authors on request.
